# The development and evaluation of an online hearing loss prevention program

**DOI:** 10.1016/j.pmedr.2023.102298

**Published:** 2023-06-25

**Authors:** Andreas Thulin, Kim Kähäri, Milijana Malmberg

**Affiliations:** a Region Västra Götaland, Habilitation & Health, Hearing Organization, Gothenburg, Sweden; b Department of Health and Rehabilitation, Institute of Neuroscience and Physiology, Sahlgrenska Academy, University of Gothenburg, Sweden

**Keywords:** Hearing loss prevention, Hearing conservation, Online hearing rehabilitation, Listening habits, Online intervention

## Abstract

Recent studies underline the need for research on approaches to encouraging healthy listening habits. This study aims to develop and evaluate an online hearing loss prevention program; and examine its influence on the participants’ use of hearing protection, hearing loss prevention knowledge, attitude towards noise, and program useability and adherence.

The study was conducted in the county of Västra Götaland, Sweden, from March through October 2021. The program included four modules focused on hearing and hearing loss prevention and was accessible to the participants for four weeks. In total, 75 participants completed the program which was evaluated using the Youth Attitude to Noise Scale and four additional self-designed questionnaires. The questionnaires were deployed before and immediately after program completion.

The findings indicate that the program may influence the participants’ attitudes toward noise and the use of hearing protection. The participants who completed the program perceived high useability and increased hearing loss prevention knowledge. This program is most needed to promote healthy hearing. Future studies should target specific groups with this program and evaluate its long-term effects.

## Highlights

• This hearing loss prevention program can effectively be provided using online services.
• Online hearing loss prevention program with potential of nation-wide accessibility.
• The program has the potential of influencing hearing protective behaviour.
• The participants’ attitudes towards noise and perceived barriers to hearing protection use were positively influenced.

## 1 Introduction

Hearing loss (HL) is one of the most common health issues and the fourth leading cause of years lived with disability worldwide (Wilson et al., 2017). Not all HL is preventable. However, noise-induced HL is, and its prevention may yield benefits in psychosocial well-being and educational achievements, and may positively impact economic independence across the life span (World Health Organization, 2021). Noise-induced HL may occur due to unsafe listening habits in both professional and recreational activities (Engdahl and Aarhus, 2021). Preventing noise-induced HL includes avoiding exposure to high sound levels and/or using hearing protection (HP) when needed. Many are unaware of the potential risks of listening at high sound levels, and such awareness is not necessarily linked to actions to preserve hearing (Matei et al., 2018, Widén, 2013). Musicians, for example, are generally aware of the dangers but may choose not to use HP due to the impact on musical performance (Matei et al., 2018). However, musicians are more likely to use HP in recreational settings (Couth et al., 2022). However, research showed that HP-use in recreational activities is predicted by HP-use in professional settings (Beach et al., 2016). Thus, HL prevention should consider unsafe listening habits in everyday life.

A recent systematic review showed that unsafe listening is highly prevalent in young individuals aged 12–34 years (Dillard et al., 2022). Experiencing HL, tinnitus and hyperacusis are common consequences of unsafe listening (Pienkowski, 2021). These consequences are highly related to negative attitudes toward noise and presumably higher HP-use (Widén, 2013). Also, personal perception and emotions related to noise, and a lack of knowledge of the consequences of unsafe listening are examples of factors shown to be crucial when deciding on HP-use (Gopal et al., 2019, Beach et al., 2012, Hunter, 2017). A population study from the United States revealed that only 8% of adults aged ≥18 use HP at loud entertainment events, with young individuals aged 18–24 years being significantly more likely to use HP than adults aged ≥ 35 years (Eichwald et al., 2018). Further on, adults experiencing HL are more likely to use HP than those without HL (Beach et al., 2016). An earlier study report younger individuals with HL being especially at risk of being afflicted with noise-induced HL due to exposure to louder volume and longer listening sessions compared to individuals without HL (Widén et al., 2018). Worsened thresholds could be restored, but repetitive exposure may cause permanent HL (You et al., 2020). Nevertheless, attitudes toward healthy hearing should be evaluated among individuals both with and without HL.

Shaping knowledge and addressing health consequences are important components when promoting health behaviour change (Loughran et al., 2021, Michie et al., 2013). For example, it may increase awareness of the risks of listening to loud music (Matei et al., 2018). Health information needs to be obtained and understood, as research has shown a relationship between general health literacy and hearing-behaviour explained by gender, educational level, and HL (especially unaddressed HL) (Clouston et al., 2017, Wells et al., 2020, Zanobini et al., 2021). Women are more likely to have higher health literacy than men (Clouston et al., 2017, Wells et al., 2020), yet contradictory, literature showed that HP is significantly used more among men than women (Beach et al., 2016). However, findings indicate that HL prevention programs (HLPP) can change audiological knowledge and listening habits, emphasizing the importance of a blended learning environment, theoretical and practical interactive sessions, personal relevance, ease of program use, and opportunities for repetition (Taljaard et al., 2013, Saunders et al., 2015). Additionally, individuals experiencing hearing-related symptoms are more likely to enroll in HLPPs (Matei et al., 2018). However, there is a need for further research and evidence to support the beneficial effects of HLPPs (Khan et al., 2018, Loughran et al., 2020).

Online interventions may improve the accessibility of hearing health care and can be a cost-effective way change of listening habits (Beukes et al., 2019, Maidment et al., 2020). Such interventions enable the repetition of information which could facilitate knowledge acquisition. Preventive information, motivation to change, and guidance are examples of actions needed to actualize the change (Laplante-Lévesque et al., 2015). Moreover, it is recommended using credible sources to provide information to initiate the use of HP (Loughran et al., 2021). Thus, addressing health-consequences using an online HLPP might be useful to improve hearing health behaviour.

Our aim was threefold: a) to develop an online HL prevention program (oHLPP), b) to investigate the program adherence, and c) to evaluate the oHLPP within the group of participants who completed the program. We wanted to investigate whether the oHLPP could increase the perceived knowledge of HL prevention among participants who completed the program and enhance their hearing protective behaviour between pre-and post-intervention, for the total group, and between subgroups related to age and HL/No HL. The participants were divided into 20–65y, representing the working-age category/professionals, and ≥ 66y representing the non-working population as 65 is the retirement age in Sweden. Recreational activities may dominate in this part of life (Vilhelmson and Thulin, 2022).

## 2 Methods and materials

The study was conducted in the county of Västra Götaland, Sweden, March–October 2021. The study was approved by the Swedish Ethical Review Authority (Dnr. 2020–01966) and conducted using an experimental design where all participants received the same intervention, and the outcomes were assessed over time.

### 2.1 Developing the online hearing loss prevention program (oHLPP)

The oHLPP was created by experienced clinically practicing and research audiologists with knowledge of online interventions and HL prevention. The program was developed using the Sweden's national Health Care Platform (HCP). The oHLPP consists of four modules, each underpinned with components and modalities aimed to increase and reinforce HL prevention knowledge (see Table 1).

**Table 1:** Illustration of modules and modalities as well as the content of the online hearing loss prevention program.

	**Module content**	**Modalities**
**Week 1**	**Anatomy and physiology:** General information about audiology and the hearing system including pathology, hearing loss, and symptoms.	
**Week 2**	**Sound, noise, and hearing protection:** Information concerning noise influence, how to listen safely, and why protecting your hearing is important.	
**Week 3**	**Hearing-related symptoms:** Information on tinnitus and hyperacusis and the impact on sleep and stress, including potential treatments.	
**Week 4**	**Rules, noise limits, and recommendations:** Information on current Swedish rules and noise regulations. Recommendations and tips to encourage healthy hearing behaviours.	

Introductory summaries and quizzes were used throughout the oHLPP except for week 1. This effectively promoted repetition of the information, which facilitates knowledge acquisition and may help identify knowledge gaps while building confidence in knowledge retention (Maidment et al., 2020, Mayer, 2017, Novacek, 2013). Furthermore, including reflection tasks may help the program users better process the information and increase their learning capacity (Mantle, 2019), and may increase their self-awareness and encourage active engagement in preventive work by empowering users to manage their behaviour (Michie et al., 2013). The oHLPP includes pictures with text throughout the program and highlights behavioural progress, not decline (Ford and Gross, 2019). In addition to addressing beliefs about consequences and emotions, pictures could also help program users maximize knowledge retention (Mayer, 2017). Videos were also created and used throughout the program to address beliefs about consequences and to visualize certain audiological concepts. The videos were subtitled to make the information readable. Examples of pure tone frequencies were included to illustrate the difference in perception between the frequencies (Saunders et al., 2015).

Each module took approximately one hour to complete, and all content was printable except the videos. To encourage program adherence, weekly notifications were sent through the HCP to inform users that new content was available. The HCP also contained a message service allowing program users to contact the research team directly with questions or feedback. After completing the oHLPP, users were encouraged to use their newly acquired hearing preventive knowledge in their everyday life by summarizing the benefits of HP-use and pointing out important take-home messages.

A pilot group of 12 participants (women, n = 7; men, n = 5) with self-reported HL, n = 2 (age 66 and 72) and without self-reported HL, n = 10 (age range 18–58, Mean = 37, SD = 13.43) evaluated the first version of the oHLPP verbally and/or by written feedback to the research team. These participants were personal acquaintances of the research group and were contacted via phone, text message, or social media. The pilot group had access to the oHLPP for two weeks and was asked to address questions regarding the content of the program, its relevance, comprehensibility, text quantity, and level of overall adversity. In addition, the online prevention program was evaluated regarding its structure, usability, length, layout, and utility.

The oHLPP was perceived as relevant and comprehensive, and the content was generally perceived as easy to understand. The pilot group had a positive view of the layout of the program and found it easy to log in and navigate through the pages. The pilot group suggested improvements such as: extending the number of videos, condensing some of the text information, including introductory text to the quizzes for the participant to better understand the purpose and having the program accessible for four weeks, with one module presented each week.

Based on these suggestions, redundant text was removed, and additional videos and introductory texts were included with the quizzes. After this revision, the new version was shared with the pilot group, and no further changes were suggested.

### 2.2 Evaluation of the oHLPP and program adherence

The inclusion criteria for the oHLPP evaluation were for the participant to be ≥ 18 years of age, and to have access to the HCP. Participation was voluntary, and the participants could at any time withdraw from the study.

First, an invitation letter and a consent form were sent to 600 hearing aid users who had been diagnosed with HL by an audiologist at the Hearing Organization, the County of Västra Götaland. Second, a volunteer sampling using advertisements through different social media- (Facebook, Instagram) and county websites were used. The participants were encouraged to visit the oHLPP program. Lastly, without using any advertisement, the oHLPP was available for those actively searching for an online program to participate in. Volunteers who returned a signed consent form with a request for additional information about the study were contacted by phone. A summary of the recruitment process can be found in Table 2.

**Table 2:** The study recruitment process for adults ≥ 18 years of age, March–October 2021, Sweden.

	Hearing aid users (from Hearing Organization)	The public (with advertisement)	The public (without any advertisement)	
Recruitment period, 2021	March to May N	May to July N	July to October N	Total N
Agreed to participate (signed a consent form)	104	104	83	291
Discontinued immediately	47	44	48	139
Answered the questionnaires pre-intervention, but failed to complete the *oHLPP*	12	36	29	77
Answered the questionnaires pre-and/or post-intervention, completed the *oHLPP*	45	24	6	75
oHLPP = online hearing loss prevention program.				

The outlines of the questionnaires deployed pre- and post-intervention can be found in Table 3, and full versions of the self-designed questionnaires and responses can be accessed through Appendix 1,2,3,4. All questionnaires were accessible online as a part of the oHLPP through the HCP to facilitate the data collection. These were mandatory for all participants, but the questionnaires pre-intervention were not forced-choice for hearing aid users, resulting in some missing measures. This was revised before the recruitment of the public group where participants had to complete initial questionnaires before getting access to the oHLPP.

**Table 3:** Summary of the questionnaires used pre-, pre- and post-, and post-intervention in the evaluation of the hearing loss prevention program for participants (≥18 years of age), recruited March–October 2021, Sweden.

**Questionnaires**	**Focus**	**Items**	**Response**	**Example**
**Pre-intervention**				
Questions on hearing (Appendix 1)	Diagnosed and/or self-reported hearing loss & hearing-related symptoms.	9	Mixed responses*	”I consider myself hearing well.”, “I’m sensitive to everyday noises which other persons don’t react to.”
Questions on knowledge (Appendix 2)	Previous knowledge about hearing loss prevention & educational level.	8	Mixed responses*	“I know exactly how to protect my hearing.”, “I’d like to learn more about hearing loss prevention.”
**Pre- and post-intervention**				
Questions on hearing protection habits (Appendix 3)	Hearing protection habits.	9	Likert scale of 0–6, 0 = Not applicable From 1 (never) to 6 (always)	“When being exposed to loud noise during my leisure time, I use hearing protection.”
Youth Attitude to Noise Scale (YANS)	Attitude toward the noise, Barriers, Norms, Intention, Control, Risk-taking, Habits, and Risk judgement.	22	Likert scale of 1–5 from 1 (totally disagree) to 5 (totally agree)	“Music is best when it’s played loud.”, “You must take risks if you want to experience something.”
**Post-intervention**				
Statements on program evaluation (Appendix 4)	The perceived increase in knowledge, influence, useability, and participation.	11	Likert scale of 1–5 from 1 (totally disagree) to 5 (totally agree)	“I believe that the program has increased my knowledge of hearing loss prevention.”, “I experience the program as useable.”

*Mixed responses include nominal and categorical response alternatives.

As the HCP requires personal data authentication, age and gender were collected through the platform. These demographics together with information on hearing aid use from the recruitment process and responses on questionnaires deployed pre-intervention (see Table 3) are presented in Table 4.

**Table 4:** Demographic data of the participants (≥18 years of age, recruited March–October 2021, Sweden) who completed the online hearing loss prevention program.

		n = 75
Gender, n (%)		
	Female	42 (56)
	Male	33
Age, years mean (SD^1^)		n = 51
	(range 20–65 years)	51 (11.4)
	Q1^2^	46
	Q2^2^	54
	Q3^2^	60
Age, years mean (SD^1^)		n = 24
	(range 66–92 years)	72 (5.2)
	Q1^2^	67
	Q2^2^	71
	Q3^2^	75
Hearing aid user, n (%)	Yes No	n = 75 45 (60) 30
Hearing loss^3^, n (%)		n = 72 (missing = 3)
	Yes	56 (78)
	No	16
Hearing-related symptoms		n = 72 (missing = 3)
Tinnitus^4^, n (%)	1–2 (never/seldom)	25
	3–5 (sometimes/often/always)	47 (65)
Hyperacusis^5^, n (%)	1–2 (never/seldom)	30
	3–5 (sometimes/often/always)	42 (58)
Education, n (%)		n = 71 (missing = 4)
	Compulsory school	8
	Secondary education	22
	Post-secondary education	41 (58)

1 Standard deviation; ^2^Q1 = the first quartile, Q2 = the second quartile, Q3 = the third quartile; ^3^Based on the answer to the statement *“I am diagnosed with hearing loss”*. ^4^Based on the answer to the statement *“I experience tinnitus that lasts for longer than 5 min”.*^5^ Based on the answer to the statement “*I am sensitive to everyday noises which other persons don’t react to*.”.

The oHLPP was accessible to the participants for as long as the data collection continued. Though designed to last four weeks, the participants themselves decided on the appropriate study pace. The research group was able to monitor the participants’ online activity through the HCP, for example when a participant activated a new module. If participants printed out the information, online monitoring was not possible.

### 2.3 Statistical analysis

Program adherence was investigated using descriptive statistics. A ranking approach (Svensson and Starmark, 2002) was used to visualise the program’s impact on HP-use habits pre- to post-intervention. All questions included in the self-designed questionnaire measuring HP-use were included. The participants who responded, “not applicable” to any of the questions were excluded from the analysis. A bootstrapping for bivariate correlation was used to estimate confidence intervals for the participants.

Due to a lack of validated questionaries measuring attitudes toward noise for an older population, the Youth Attitude to Noise Scale (YANS) was used even though validated for individuals below thirty years of age (Widén, 2013). The subscales were interpreted as described elsewhere (Widén, 2013). In another study, the YANS successfully underpinned the development and evaluation of a questionnaire assessing knowledge, attitudes, and behaviours to HL prevention among participants 18–80 years old (Saunders et al., 2014). These results add value to using the YANS for the total sample in the current study. The participants were also analysed in subgroups addressing age and being diagnosed/not with HL. The Wilcoxon Signed-Rank test was used to compare the median differences pre- to post-intervention for the total sample and subgroups, as the data are considered ordinal. Mann-Whitney U was used to investigate the differences between the subgroups. Lastly, descriptive statistics were used post-intervention to describe the participants’ perceived knowledge and the useability of the oHLPP. The significance level was set to 0.05 (5%).

## 3 Results

### 3.1 The program adherence and target population

The demographics and hearing-related information of the participants collected using questionnaires on hearing and knowledge pre-intervention (see Appendix 1 and 2) can be found in Table 4.

### 3.2 Evaluation of the oHLPP

A total of 75 participants completed the oHLPP. Those who did not respond to questionnaires pre-and/or post-participation were excluded from that specific analysis. Accordingly, 70 participants evaluated HP-use, 69 the outcomes measured with YANS, and 67 evaluated perceived knowledge and the usability of the oHLPP.

**Fig. 1:**
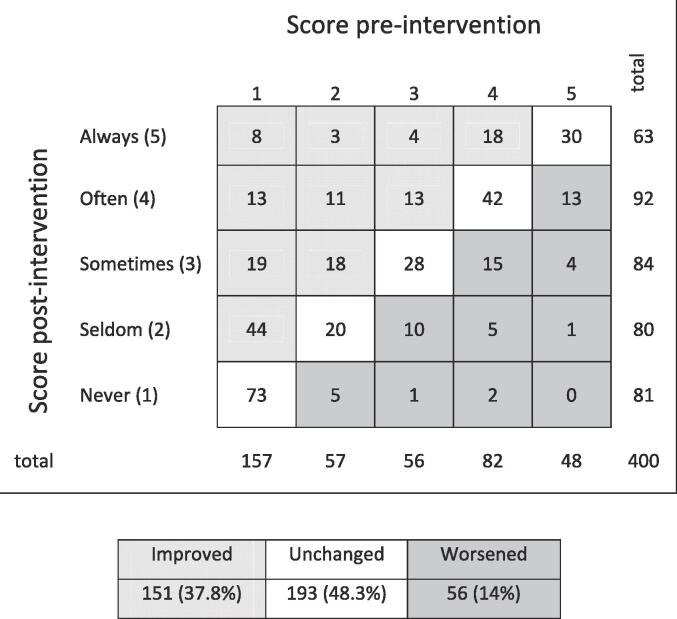
Case summary of change for the self-designed questionnaire, measuring hearing protection usage pre- to post-intervention for participants (≥18 years of age, recruited March–October 2021, Sweden) who completed the online hearing loss prevention program.

Fig. 1 illustrates a summary of all responses regarding the likeliness of HP-usage in different loud environments (see Appendix 3). Each case represents participants’ responses to one of the nine questions pre- to post-intervention. For example, the grey lined area of Fig. 1 shows that in 13 cases, the participants who responded that they *Never* (Wilson et al., 2017) use protection in the stated situation changed to *Often* (Matei et al., 2018). The participants who responded that they *Seldom/Never* used HP (see Fig. 1) were more likely to increase usage. In total 37.8% of the responses improved after oHLPP completion, 48.3% were unchanged, and 14% worsened (95% CI 0.6–0.7).

Statistically significant differences were found pre- to post-intervention in subscales “Norms” and “Intention” for the total YANS scale and all subgroups (Table 5). No statistically significant improvement was seen for the subscale “Risk judgement”. The subscales “Risk-taking” and “Habits” showed statistically significant improvements for the total sample and the subgroups “20–65y” and “No HL”. The “Attitude” subscale showed improvements in all subgroups except for the subgroup “≥66y”. On the other hand, this subgroup showed statistically significant improvements for the subscale “Barriers”. No statistically significant differences were found between the subgroups “20–65y” and “≥66y”, nor between the subgroups “HL” and “No HL”.

**Table 5:** Means, standard deviations (SD), medians, 1st (Q1), and the 3rd (Q3) quartile, and Wilcoxon Signed-Rank test pre- to post-intervention for the Youth Attitude to Noise Scale subscales presented with *p*-values. The results concern the participants (≥18 years of age, recruited March–October 2021, Sweden) who completed the online hearing loss prevention program.

	**Demographics**		**Attitude**	**Barriers**	**Norms**	**Intention**	**Control**	**Risk-taking**	**Habits**	**Risk judgement**
**Total (n = 69)**	Female 58% HL 77% Tinnitus^#^ 68% Hyperacusis^#^ 61% Sec. educ. 32% Post-sec. educ. 59%	**Mean (SD)**	−0.21 (0.043)	−0.17 (0.78)	0.49 (1.11)	0.57 (0.99)	0.22 (0.89)	−0.29 (1.20)	0.41 (1.25)	0.12 (1.02)
		**Median**	−0.27	−0.20	0.00	0.00	0.00	0.00	0.00	0.00
		**Q1 to Q3**	−0.45 to 0.05	−0.70 to 0.20	0.00 to 1.00	0.00 to 1.00	0.00 to 1.00	−1.00 to 0.00	0.00 to 1.00	0.00 to 1.00
		***p-*value**	<0.001*	0.042*	<0.001*	<0.001*	0.056	0.022*	0.010*	0.168
**20-65y (n = 48)**	Female 62% HL 67% Tinnitus^#^ 67% Hyperacusis^#^ 69% Sec. educ. 31% Post-sec. educ. 60%	**Mean (SD)**	−0.27 (0.40)	−0.09 (0.76)	0.50 (1.15)	0.56 (0.94)	0.29 (0.94)	−0.40 (1.14)	0.40 (1.32)	0.13 (1.12)
		**Median**	−0.36	0.00	0.00	0.00	0.00	−0.50	0.00	0.00
		**Q1 to Q3**	−0.55 to 0.00	−0.40 to 0.20	0.00 to 1.00	0.00 to 1.00	0.00 to 1.00	−1.00 to 0.00	0.00 to 1.00	0.00 to 1.00
		***p-*value**	<0.001*	0.268	0.005*	<0.001*	0.040*	0.016*	0.045*	0.220
**≥66y (n = 21)**	Female 43% HL 100% Tinnitus^#^ 62% Hyperacusis^#^ 71% Sec. educ. 29% Post-sec. educ. 52%	**Mean (SD)**	−0.09 (0.49)	−0.37 (0.80)	0.48 (1.03)	0.57 (1.12)	0.05 (0.74)	−0.05 (1.32)	0.43 (1.12)	0.10 (0.77)
		**Median**	−0.09	−0.40	0.00	0.00	0.00	0.00	0.00	0.00
		**Q1 to Q3**	−0.36 to 0.09	−1.10 to 0.20	0.00 to 1.00	0.00 to 1.00	0.00 to 0.50	−1.00 to 0.50	0.00 to 1.00	0.00 to 0.50
		***p-*value**	0.234	0.049*	0.033*	0.033*	0.763	0.595	0.097	0.564
**HL (n = 53)**	Female 45% Tinnitus^#^ 75% Hyperacusis^#^ 60% Sec. educ. 32% Post-sec. educ. 52%	**Mean (SD)**	−0.17 (0.46)	−0.17 (0.84)	0.43 (1.14)	0.53 (1.05)	0.13 (0.83)	−0.17 (1.22)	0.36 (1.35)	0.06 (1.05)
		**Median**	−0.09	−0.20	0.00	0.00	0.00	0.00	0.00	0.00
		**Q1 to Q3**	−0.45 to 0.90	−0.80 to −0.20	0.00 to 1.00	0.00 to 1.00	0.00 to 1.00	−1.00 to 0.50	0.00 to 1.00	0.00 to 0.00
		***p-*value**	0.008*	0.087	0.010*	<0.001*	0.263	0.162	0.057	0.405
**No HL (n = 16)**	Female 94% Tinnitus^#^ 33% Hyperacusis^#^ 48% Sec. educ. 25% Post-sec. educ. 75%	**Mean (SD)**	−0.37 (0.31)	−0.18 (0.58)	0.69 (1.01)	0.69 (0.79)	0.50 (1.03)	−0.69 (1.08)	0.56 (0.89)	0.31 (0.95)
		**Median**	−0.36	−0.10	1.00	0.50	0.00	−1.00	0.00	0.00
		**Q1 to Q3**	−0.61 to −0.09	−0.40 to 0.15	0.00 to 1.75	0.00 to 1.00	0.00 to 1.00	1.00 to 0.00	0.00 to 1.00	0.00 to 1.00
		***p-value***	0.002*	0.235	0.022*	0.009*	0.034*	0.035*	0.030*	0.212

*p < 0.05; HL = Hearing loss; ^#^Experiencing tinnitus/hyperacusis sometimes/often/always; Sec. educ. = Secondary education; Post-sec. educ. = Post-secondary education. Demographic data for the total sample and different subgroups is presented with %.

When evaluating the oHLPP (see Appendix 4) the participants generally found that the program positively influenced their knowledge, and they also responded positively to the program’s useability and the overall attitude towards participation in the oHLPP. Based on descriptive analyses the evaluation of the oHLPP was similar across all participants regardless of their age and hearing status.

## 4 Discussion

The study findings indicate that the oHLPP may positively influence perceived knowledge and attitudes towards noise and could increase HP-use for the participants. The evaluation of the oHLPP reveals positive experiences regarding the participation and program’s useability.

## 5 Program adherence and target population

One of the general purposes and goals of HL prevention is to access individuals with harmful listening habits (World Health Organization, 2021). Certain factors seem to be indicative of which groups are at risk, where individuals with lower socio-economic status, lower education, and higher age are at greater risk (Zanobini et al., 2021). One challenge in creating oHLPPs is to make them desirable for large groups of people and to access these at-risk groups. The participants of the current oHLPP generally had a high level of education, were mostly women, and diagnosed with HL and/or experienced hearing-related problems. As individuals with higher education have a higher level of health literacy (Zanobini et al., 2021), this group might be more attentive toward potential risks and more willing to act on them. Women tend to have higher health literacy (Clouston et al., 2017, Zanobini et al., 2021), but on the other hand, men tend to use HP more often than women (Beach et al., 2016). The latter could perhaps be explained by differences in occupation, where some noisy occupations are still male-dominated, for example, the manufacturing industry. Nevertheless, males have shown to have a higher risk of noise-induced HL regardless of age (Wang et al., 2021).

Furthermore, as experiencing HL influences HP-use it might be that HL, in relation to hearing-related symptoms (Beach et al., 2016, Widén et al., 2011), also motivates engagement in oHLPP. Hearing related symptoms have shown to represent internal cues to take action, causing individuals to accept an invitation to participate in HL prevention programs (Saunders et al., 2012). In addition, it might be that those without diagnosed HL who answered the questionnaires pre-intervention but failed to complete the oHLPP had insufficient knowledge of health consequences related to HL. This has previously shown to result in a lack of concern for hearing health (Hunter, 2018). Consequently, being diagnosed with HL is not indicative of the general population, and the target demographic was not reached using this paper’s recruitment strategy.

In the present study, social media was used to recruit participants from the general population. To reach participants who could benefit from the knowledge but have no interest in similar programs, other approaches might be better suited, like workplace training seminars (Stephenson et al., 2011) or the inclusion of HL prevention in the school curriculum (Griest et al., 2007). For students attending schools where the intended profession is prone to noise-induced HL, HL preventive knowledge should be included.

### 5.1 Evaluation of the oHLPP

The evaluation of the present oHLPP showed that the participants who completed the oHLPP perceived increased knowledge as well as increased motivation to assimilate preventive measures. In total 37.8% of the participants intended to use hearing protection to a larger extent post-intervention, even though a majority presented HP-use pre-intervention. These results are in line with a previous study revealing that an on-site HLPP influenced HP-usage for the better (Folmer et al., 2012). Nevertheless, 48.3% of the participants in the current study showed no change in their habits and 14% reported HP-use to a lesser extent after completing the program. Previous research showed that intention is not always indicative of actual behavioural change, and 50% fail to act on their intentions (Sheeran and Webb, 2016). Thus, the questionnaire on HP used in the current study might be less sensitive for detecting changes in HP-habits.

The YANS results revealed successful influence for the subscales “Attitude”, “Norms”, and “Intention” for the total sample and in most subgroups. This is interesting as the participants of the oHLPP mostly consist of individuals experiencing HL or hearing-related symptoms, and hence already may have negative attitudes towards noise and are presumably HP-users (Widén, 2013, Beach et al., 2016). Yet, it might be that the participants are generally positive pre- to post-intervention simply by being enrolled in a research study (Linde et al., 2011). Furthermore, no change in “Attitude” was shown in the older subgroup (≥66y), which could indicate that the oHLPP had a greater impact on younger individuals’ listening habits. It may be that the younger population is generally more exposed to high sound levels and by that more motivated to protect their hearing. It may also be due to the YANS being more suited for changes in a younger population, even among those 30 to 65 years (Widén, 2013). The results may also indicate that the older group (≥66y) experienced more positive attitudes toward HP-use pre-intervention (Beach et al., 2016).

The subscale “Risk judgement” showed no statistically significant improvements. This could indicate that the oHLPP is not sufficiently addressing the potential risks of noise-induced HL for a change to occur, or that the participants initially already are aware of the potential risks. Also, analysis of the complete sample showed a significant improvement for the “Barriers” subscale as well as the subgroup “≥66y”. Addressing barriers to HP-use is important when creating an oHLPP to motivate behavioural change (Loughran et al., 2021), and even though addressed throughout the current oHLPP, more emphasis on barriers to HP-use in future oHLPPs could be needed.

Furthermore, the participants without HL improved in more subscales than individuals with HL. This might be due to the eventual pre-existing knowledge on HL consequences for individuals with HL (Hunter, 2017). Nevertheless, many of the participants in this subgroup (No HL) are experiencing hearing-related symptoms such as tinnitus, which is related to negative attitudes towards noise and higher HP-use (Widén, 2013).

Finally, the oHLPP positively influenced the participants perceived knowledge and comprehension of HL prevention. These results are in line with previous research identifying improvements in knowledge when offering hearing education intervention (Saunders et al., 2015, Saunders et al., 2014). It might lead to higher HP-use (Saunders et al., 2015), however, to increase knowledge and awareness of the potential risks of listening at high sound levels, the knowledge needs to be relevant and accessible to the consumers (Hunter, 2018).

### 5.2 Study limitations

One of the study limitations is that the study sample had a large age range and a high level of heterogeneity, making in-group comparisons subtle. Also, the participants recruited from the public self-reported diagnosed HL. Self-reported hearing measures are shown to be insufficiently sensitive to predict the incidence of HL (Kiely et al., 2012). In addition, no information regarding hearing aid usage was collected, which could have affected the findings.

Another limitation is that the major part of the participants in the current study are not covered by the validation of YANS (Widén, 2013), and the results should be interpreted with this in mind. Lastly, it is important to note certain characteristics of the method used to visually summarize the participants’ progress in HP use (Svensson and Starmark, 2002). Even if the individual believed his or her habits had changed during participation, or after, the questionnaire does not allow an increase from 5 or a decrease from 1. This poses a ceiling and floor effect in the results.

### 5.3 Future perspectives

Together with regulations, societal norms, and organisational work on a top-down level, HL prevention using online resources may empower and target a broad spectrum of people on different levels in society. The current oHLPP is therefore one of the puzzles needed to promote healthy hearing. Future studies should explore the attractiveness of the program to younger individuals (Harris et al., 2017) and target specific groups, for example, compulsory school students or high-risk individuals. The oHLPP will be followed up at six- and twelve-months post-intervention to investigate the long-term effects, and this will be presented in a future paper.

## 6 Conclusions

The results of the current study indicate that an oHLPP that is accessible from a credible source attracts mostly highly educated individuals and those experiencing hearing-related symptoms. Completing the oHLPP may influence perceived knowledge of HL prevention, regardless of age or experiencing HL/no HL. Moreover, it may influence attitudes toward loud noise, especially among the younger participants. Further, it may have a positive impact on the intention to use HP. Developing the oHLPP enables encouraging healthy hearing on a national level.

**Source of Funding:** This study was sponsored by the Interreg Sweden-Norway, European Regional Development Fund (ref 2018–00509), and by Hearing Organization in Region Västra Götaland, Sweden.

## CRediT authorship contribution statement

**Andreas Thulin:** Conceptualization, Methodology, Writing – original draft, Software, Investigation, Writing – review & editing, Visualization, Formal analysis. **Kim Kähäri:** Conceptualization, Methodology, Writing – original draft, Supervision. **Milijana Malmberg:** Conceptualization, Methodology, Writing – original draft, Software, Investigation, Writing – review & editing, Visualization, Supervision, Data curation, Project administration, Funding acquisition.

## Declaration of Competing Interest

The authors declare that they have no known competing financial interests or personal relationships that could have appeared to influence the work reported in this paper.

## Figures and Tables

**Appendix 1:** Questions and responses on hearing, pre-intervention, answered by the participants (≥18 years of age, recruited March–October 2021, Sweden) who completed the online hearing loss prevention program (n = 72).

**Questions**	**Response options**	**n (%)**
I consider myself hearing well.	Yes No	14 (19.4) 58 (80.6)
I am diagnosed with hearing loss.	Yes No	56 (77.8) 16 (22.2)
I experience difficulties hearing well when several persons speak at the same time.	Never Seldom Sometimes Often Always	4 (5.6) 4 (5.6) 21 (29.2) 23 (31.9) 20 (27.8)
I experience tinnitus that lasts for longer than 5 min.	Never Seldom Sometimes Often Always	16 (22.2) 9 (12.5) 12 (16.7) 9 (12.5) 26 (36.1)
I experience tinnitus after listening to loud noises (for example after a concert).	Never Seldom Sometimes Often Always	20 (27.8) 16 (22.2) 18 (25.0) 5 (6.9) 13 (18.1)
I experience a sensation of blocked ears after listening to loud noises (for example after a concert).	Never Seldom Sometimes Often Always	18 (25.0) 22 (30.6) 19 (26.4) 7 (9.7) 6 (8.3)
I am sensitive to everyday noises which other persons don’t react to.	Never Seldom Sometimes Often Always	13 (18.1) 17 (23.6) 23 (31.9) 14 (19.4) 5 (6.9)
Sounds other persons experience as fine, sound broken, unclear, and distorted to me.	Never Seldom Sometimes Often Always	21 (29.2) 15 (20.8) 25 (34.7) 8 (11.1) 3 (4.2)
I experience sound fatigue in my ears/head when having worked or been exposed to loud noises during the day.	Never Seldom Sometimes Often Always	9 (12.5) 16 (22.2) 15 (20.8) 20 (27.8) 12 (16.7)

The responses are presented with n (%).

**Appendix 2:** Questions and responses regarding previous knowledge on hearing loss prevention and personal educational level, pre-intervention, answered by the participants (≥18 years of age, recruited March–October 2021, Sweden) who completed the online hearing loss prevention program (n = 71).

**Questions**	**Response options**	**n (%)**
I know exactly how to protect my hearing.	Yes No	25 (35.2) 46 (64.8)
I know which sounds are harmful.	Yes No	40 (56.3) 31 (43.7)
I’ve learned how to protect my hearing through education at the compulsory school level.	Yes No	9 (12.7) 62 (87.3)
I’ve learned how to protect my hearing through education at the secondary education level.	Yes No	8 (11.3) 63 (88.7)
I’ve learned how to protect my hearing through education at my workplace.	Yes No	20 (28.2) 51 (71.8)
I’ve learned how to protect my hearing by finding the information myself.	Yes No	39 (54.9) 32 (45.1)
I’d like to know more about hearing loss prevention.	Yes No	46 (64.8) 25 (35.2)
My highest educational level (finished/in progress).	Compulsory school Secondary education Post-secondary education	8 (11.3) 22 (31.0) 41 (57.7)

The responses are presented with n (%).

**Appendix 3:** Questions and responses regarding hearing protection habits pre- and post-intervention answered by the participants (≥18 years of age, recruited March–October 2021, Sweden) who completed the online hearing loss prevention program (n = 70).

	**Questions**	**Response options**	**Pre-intervention, n (%)**	**Post-intervention, n (%)**
When being exposed to loud noise when working/attending school, I use hearing protection.		Not applicable Never Seldom Sometimes Often Always	26 (37.1) 13 (18.6) 5 (7.1) 9 (12.9) 13 (18.6) 4 (5.7)	24 (34.3) 7 (10.0) 7 (10.0) 10 (14.3) 15 (21.4) 7 (10.0)
When being exposed to loud noise during my leisure time, I use hearing protection.		Not applicable Never Seldom Sometimes Often Always	9 (12.9) 11 (15.7) 9 (12.9) 11 (15.7) 25 (35.7) 5 (7.1)	10 (14.3) 3 (4.3) 9 (12.9) 12 (17.1) 26 (37.1) 10 (14.3)
When being exposed to impulse noises (e.g., from hammer blows, firecrackers, and gun discharges), I use hearing protection.		Not applicable Never Seldom Sometimes Often Always	9 (12.9) 11 (15.7) 13 (18.6) 9 (12.9) 18 (25.7) 10 (14.3)	6 (8.6) 4 (5.7) 14 (20.0) 18 (25.7) 17 (24.3) 11 (15.7)
When being in the environment of/when using loud machines or vehicles (e.g., using a lawn mower, MC, motocross), I use hearing protection.		Not applicable Never Seldom Sometimes Often Always	11 (15.7) 12 (17.1) 7 (10.0) 6 (8.6) 18 (25.7) 16 (22.9)	8 (11.4) 5 (7.1) 9 (12.9) 13 (18.6) 11 (15.7) 24 (34.3)
When attending events with loud noises (e.g., sporting events, concerts, festivals), I use hearing protection.		Not applicable Never Seldom Sometimes Often Always	14 (20.0) 16 (22.9) 12 (17.1) 12 (17.1) 8 (11.4) 8 (11.4)	11 (15.7) 6 (8.6) 8 (11.4) 19 (27.1) 14 (20.0) 12 (17.1)
When attending restaurants, pubs, or similar events where loud noises occur, I use hearing protection.		Not applicable Never Seldom Sometimes Often Always	4 (5.7) 52 (74.3) 9 (12.9) 3 (4.3) 2 (2.9) 0 (0)	7 (10.0) 27 (38.6) 23 (32.9) 7 (10.0) 4 (5.7) 2 (2.9)
When working out with loud noises, I use hearing protection.		Not applicable Never Seldom Sometimes Often Always	23 (32.9) 29 (41.4) 5 (7.1) 6 (8.6) 4 (5.7) 3 (4.3)	33 (47.1) 10 (14.3) 13 (18.6) 4 (5.7) 6 (8.6) 4 (5.7)
When playing or singing in a band/choir/orchestra, I use hearing protection.		Not applicable Never Seldom Sometimes Often Always	52 (74.3) 13 (18.6) 1 (1.4) 2 (2.9) 0 (0) 2 (2.9)	50 (71.4) 10 (14.3) 2 (2.9) 1 (1.4) 4 (5.7) 3 (4.3)
When listening to loud noises through speakers, I use hearing protection.		Not applicable Never Seldom Sometimes Often Always	24 (34.3) 36 (51.4) 3 (4.3) 4 (5.7) 2 (2.9) 1 (1.4)	24 (34.3) 23 (32.9) 10 (14.3) 5 (7.1) 3 (4.3) 5 (7.1)

The responses are presented with n (%).

**Appendix 4:** Statements evaluating the experience of the online hearing loss prevention program, post-intervention, answered by the participants (≥18 years of age, recruited March–October 2021, Sweden) who completed the online hearing loss prevention program. Response options for each statement from 1 = totally disagree to 5 = totally agree, presented with means, standard deviations (SD) and medians for the total sample (n = 67) and different subgroups.

**Statements**		**Total sample**	**20-65y (n = 46)**	**≥66y (n = 21)**	*HL (n = 51)	**No HL (n = 16)**
I believe that the program has increased my knowledge of hearing loss prevention.	Mean (SD) Median	4.16 (0.81) 4.00	4.28 (0.87) 4.50	3.90 (0.54) 4.00	4.10 (0.78) 4.00	4.38 (0.89) 5.00
I believe that the program has increased my comprehension of the hearing loss prevention.	Mean (SD) Median	3.99 (0.90) 4.00	4.09 (0.94) 4.00	3.76 (0.77) 4.00	3.90 (0.86) 4.00	4.25 (1.00) 4.50
Attending the program has made me more equipped regarding the knowledge and comprehension of hearing loss prevention.	Mean (SD) Median	3.94 (0.95) 4.00	4.09 (1.01) 4.00	3.62 (0.74) 4.00	3.78 (0.92) 4.00	4.44 (0.89) 5.00
I experience the program as useable.	Mean (SD) Median	4.13 (1.03) 4.00	4.20 (0.77) 4.00	4.00 (1.14) 4.00	4.08 (1.00) 4.00	4.31 (1.14) 5.00
I perceive the program as being straightforward and relevant to use in general.	Mean (SD) Median	4.21 (0.81) 4.00	4.24 (0.77) 4.00	4.14 (0.91) 4.00	4.20 (0.80) 4.00	4.25 (0.86) 4.00
It was easy for me to log in and start taking part in the program.	Mean (SD) Median	4.36 (0.88) 5.00	4.46 (0.75) 5.00	4.14 (1.11) 5.00	4.29 (0.92) 5.00	4.56 (0.73) 5.00
I experience the program as easy to navigate.	Mean (SD) Median	4.07 (0.97) 4.00	4.07 (0.90) 4.00	4.10 (1.14) 5.00	3.98 (1.05) 4.00	4.38 (0.62) 4.00
I received clear and understandable instructions on how to use the program.	Mean (SD) Median	4.21 (0.85) 4.00	4.17 (0.85) 4.00	4.29 (0.85) 5.00	4.12 (0.84) 4.00	4.50 (0.82) 5.00
I enjoyed partaking in the program in general.	Mean (SD) Median	3.94 (0.92) 4.00	4.09 (0.94) 4.00	3.62 (0.81) 4.00	3.90 (0.86) 4.00	4.06 (1.12) 4.00
I am positive on having taking part in the program.	Mean (SD) Median	4.37 (0.83) 5.00	4.41 (0.83) 5.00	4.29 (0.85) 4.00	4.31 (0.84) 5.00	4.56 (0.81) 5.00
I would recommend the program to other people.	Mean (SD) Median	4.24 (0.94) 4.00	4.26 (1.00) 5.00	4.19 (0.81) 4.00	4.18 (0.91) 4.00	4.44 (1.03) 5.00

## Data Availability

Data will be made available on request.
